# Impaired Circulating Angiogenic Cells Mobilization and Metalloproteinase-9 Activity after Dynamic Exercise in Early Metabolic Syndrome

**DOI:** 10.1155/2015/920356

**Published:** 2015-10-18

**Authors:** Natalia G. Rocha, Allan R. K. Sales, Leticia A. Penedo, Felipe S. Pereira, Mayra S. Silva, Renan L. Miranda, Jemima F. R. Silva, Bruno M. Silva, Aline A. Santos, Antonio C. L. Nobrega

**Affiliations:** ^1^Laboratory of Exercise Sciences, Department of Physiology and Pharmacology, Fluminense Federal University, 24210-130 Niterói, RJ, Brazil; ^2^Department of Physiology, Section of Exercise Physiology, Federal University of São Paulo, 04023-062 São Paulo, SP, Brazil

## Abstract

Increased levels of adhesion molecules or metalloproteinases (MMPs) may indicate endothelial dysfunction. Exercise mobilizes circulating angiogenic cells (CACs) from bone marrow in healthy subjects, improving vascular function. However, it is unclear whether this mechanism is preserved in the early stages of metabolic syndrome (early MetS). We aimed to evaluate the acute effects of exercise on adhesion molecules, angiogenic factors, MMPs, and CACs in early MetS. Fifteen subjects with early MetS and nine healthy controls underwent an exercise session and a nonexercise session, randomly. Adhesion molecules, angiogenic factors, CACs, and MMPs were evaluated before and after exercise or nonexercise sessions. At baseline, levels of sE-selectin, sICAM-1, and MMP-9 were higher in early MetS than in controls (*P* ≤ 0.03). After exercise, sE-selectin, sICAM-1, and MMP-9 levels were still higher in early MetS (*P* < 0.05). Subjects with early MetS presented less CACs (*P* = 0.02) and higher MMP-9 activity (*P* ≤ 0.04), while healthy controls presented higher MMP-2 activity after exercise. There was no difference between moments in nonexercise session (*P* > 0.05). In conclusion, subjects with early MetS already presented impaired endothelial function at rest along with a decrease in CACs and an increase in MMP-9 activity in response to exercise.

## 1. Introduction

Cardiovascular disease is the major cause of death worldwide [1]. Mechanisms underlying atherosclerosis are tightly related to the presence of cardiometabolic risk factors. The incidence of metabolic syndrome (MetS) has been increasing in the global population [2]. MetS is characterized by a cluster of metabolic disorders, including dysglycemia, dyslipidemia, obesity, and hypertension [3]. In the early stages of MetS development (early MetS), when no chronic diseases are yet present and no drug therapy has been used, it may be possible to determine an increased risk of atherogenesis by assessing endothelial dysfunction [4].

The intact endothelium prevents leukocyte migration into the vessel wall. This leukocyte interaction with the endothelium is regulated by the expression of cell adhesion molecules, such as endothelial leukocyte adhesion molecule (E-selectin), intercellular adhesion molecule-1 (ICAM-1), and vascular cell adhesion molecule-1 (VCAM-1) [5]. Imbalance expression of cell adhesion molecules is referred to as endothelial dysfunction [6] and is usually present in patients with MetS [7].

Circulating angiogenic cells (CACs) are usually recruited from the bone marrow to peripheral blood promoting neovasculogenesis, maintaining vascular integrity. Despite being controversial, studies have shown associations between chronic diseases, such as hypertension [8], diabetes [9], and dyslipidemia [10], and the number/functionality of CACs. Jialal et al. [11] showed a reduced number of CACs along with impaired functionality in subjects with MetS, who were using antihypertensive drugs. However, some drugs, such as antihypertensives and statins, are already known to influence the number and/or functionality of CACs [12, 13]. Thus, whether the number of CACs is already altered in drug naïve subjects with MetS is unclear.

Acute exercise provokes a transient inflammatory response through the increase in the amounts of several cytokines, angiogenic factors [14], and matrix metalloproteinases (MMPs) in the circulation [15]. MMP-2 and MMP-9 are related to inflammation, angiogenesis, wound healing, and cell migration, including CACs migration from the bone marrow to peripheral blood [16]. Subjects with established MetS-related diseases present high levels of proinflammatory markers and MMP-9 [16]. In addition, a maximal aerobic exercise seems to increase CACs in patients with coronary artery disease but less than in healthy subjects [17]. However, the acute effects of exercise on CACs and MMPs in subjects with MetS and without chronic diseases are still unknown.

This study aimed to evaluate the effects of a single bout of exercise on adhesion molecules, on angiogenic factors, on CACs, and on MMPs in subjects with early MetS. We hypothesized that subjects with early MetS, that is, free of overt disease and without using medications, already present an impaired endothelial function at baseline along with an altered response of angiogenic factors, CACs, and MMPs to exercise.

## 2. Materials and Methods

### 2.1. Ethical Approval

This study protocol was approved by the ethical committee of Antonio Pedro University Hospital, according to the latest revision of the Declaration of Helsinki. All subjects gave written informed consent before their participation in the study.

### 2.2. Subjects

Subjects were recruited through advertisements at the university and in local newspapers. Twenty-four subjects were enrolled, fifteen subjects with early MetS (MetS group, age: 37 ± 2 years old) and nine healthy subjects (controls) with none of the five criteria for MetS (healthy group, age: 33 ± 3 years old). The MetS group presented at least three of the following five criteria defined by the American Heart Association [3]: waist circumference >90 cm (men) or >80 cm (women); systolic blood pressure ≥130 mmHg and/or diastolic blood pressure ≥85 mmHg; fasting glucose ≥5.6 mmol·L^−1^; triglycerides ≥1.7 mmol·L^−1^; high-density lipoprotein cholesterol (HDL-c) <1 mmol·L^−1^ (men) or <1.3 mmol·L^−1^ (women). Other inclusion criteria included the absence of any diagnosed disease, no recent infection, no medication, nonsmoker, woman with regular menstrual cycle, and sedentary lifestyle (not attended exercise program lasting ≥30 min, three times per week during the last three months). Women had regular menstrual cycles and were evaluated in the early follicular phase (up to 5th day of menstrual cycle). The eligibility requirements were determined through clinical history assessment, physical examination, blood pressure measurement, biochemical blood analyses, resting electrocardiogram, and peak cardiopulmonary exercise testing.

### 2.3. Biochemical Blood Analyses

Blood was drawn from an anterior cubital vein in the morning after a 12-hour overnight fast. Cholesterol and its subfractions (HDL-c and low-density lipoprotein (LDL-c)) as well as triglycerides and glucose were determined using enzymatic colorimetric methods. Plasma insulin was measured by electrochemiluminescence immunoassay. Insulin resistance was estimated using the homeostasis model assessment (HOMA-IR) derived from fasting glucose and insulin concentrations [18]. Total leukocyte count was measured by an electronic counter, the HST-302N system.

### 2.4. Clinical Evaluation

A physician performed the evaluation, including clinical history assessment and resting electrocardiogram (CardioCare 2000; Bionet, Tustin, CA, USA). Resting blood pressure measurements were performed twice, one on each arm, on two separate days in the upright sitting position. Recordings were made under quiet and temperature controlled (approximately 24°C) conditions. An appropriately sized cuff (cuff bladder encircling at least 80% of the arm) was used.

### 2.5. Physical Examination

Anthropometric variables, such as weight and height, were measured using a medical beam balance (Welmy; Santa Bárbara d'Oeste, SP, Brazil). Body mass index (BMI) was calculated as weight (kg) divided by the square of the height (m). Waist circumference was considered the midpoint between the iliac crest and the last floating rib (XII rib).

### 2.6. Cardiopulmonary Exercise Testing

Subjects underwent a cardiopulmonary exercise test, performed until fatigue on a cycle ergometer (CG400 model, Inbrasport; Porto Alegre, RS, Brazil). The protocol was developed according to predicted maximal exercise capacity. Subjects were verbally encouraged to exercise until exhaustion in order to reach volitional fatigue at approximately 10 min of testing. Ventilation, oxygen uptake, and carbon dioxide output were determined with each breath (CPX Ultima Gas Exchange System, Medgraphics Corp.; St. Paul, MN, USA). An electrocardiogram was monitored through 12 leads (Welch Allyn CardioPerfect Workstation, Welch Allyn; Skaneateles Falls, NY, USA), and perceived exertion was verified every minute using the 0–10 Borg scale. Breath-by-breath ventilation and expired gas data were averaged to 20 s to identify the peak oxygen consumption (VO_2peak_), which was considered the highest value of oxygen uptake recorded during exercise. Ventilatory threshold was identified by combination of the following methods: (1) inflection of ventilation versus time curve and (2) consistent increase in the ventilatory equivalent of oxygen (VE/VO_2_) without a concomitant increase ventilatory equivalent of carbon dioxide (VE/VCO_2_).

### 2.7. Experimental Protocol

On two separate days, at least two days apart, subjects from both the healthy and MetS groups underwent the exercise session and nonexercise session in a random order. Adhesion molecules, MMP-9 (*n* = 24), and CACs (*n* = 18) were evaluated before and 10 min after the exercise or nonexercise session. During the nonexercise session, subjects sat still on the cycler ergometer for the same period of time as the exercise session. These experimental sessions were always conducted at the same time of the day after a 1-hour fast. Participants were also given standard feeding orientations for the previous day and asked to abstain from caffeine and alcohol consumption and physical exercise for at least 48 h.

### 2.8. Exercise Session

An individualized continuous submaximal bout of exercise was performed for 40 min on a cycle ergometer (CG400 model, Inbrasport; Porto Alegre, RS, Brazil) at an intensity corresponding to 80% of the ventilatory threshold, which was observed in the previous peak cardiopulmonary exercise test. This exercise was preceded by a warm-up of five minutes, pedaling at 30 W, followed by five minutes of recovery pedaling at 30 W. Breath-by-breath ventilation and expired gas were recorded throughout the exercise bout by a digital metabolic analyzer (CPX Ultima Gas Exchange System, Medgraphics Corp.; St. Paul, MN, USA), which was linked to a computer for data recording and offline analysis.

### 2.9. Concentration of Adhesion Molecules, MMP-9, and Angiogenic Factors

The levels of sE-selectin, sICAM-1, sVCAM-1, MMP-9, vascular endothelial growth factor (VEGF), granulocyte-colony stimulating factor (G-CSF), and granulocyte macrophage-colony stimulating factor (GM-CSF) were determined using a multiplex sandwich immunoassay that was performed using a Luminex 200 (Luminex; Austin, USA) and xMAP technology [19, 20]. In this assay, the specific antibody was covalently coupled to Luminex microspheres and uniquely labeled with a fluorescent dye. Briefly, the microspheres were incubated overnight with standards, controls, and serum samples in a 96-well microliter filter plate for duplicate determination. After washing the wells, a mixture of the relevant biotinylated detection antibodies was added and incubated for 30 min at room temperature. Streptavidin-phycoerythrin was then added for an additional 30 min. The beads were finally washed twice, resuspended in buffer, and analyzed by Exponent software according to the manufacturer's instructions. The results are reported as the means of the duplicates.

### 2.10. Circulating Angiogenic Cells

Peripheral blood mononuclear cells (PBMC) were isolated from the blood by Ficoll density-gradient centrifugation, according to the manufacturer's instructions. After the isolation, 5 × 10^6^ cells were incubated with 10 *μ*L of CD34-FITC (BD Biosciences; Franklin Lakes, NJ, USA), 6 *μ*L of CD133-PE (Miltenyi; Bergisch Gladbach, North Rhine-Westphalia, Germany), and 10 *μ*L of VEGFR2-APC (R&D Systems; Minneapolis, MN, USA). All antibodies were added directly to the cell suspension and kept in the dark at 4°C for 40 min. Cells were washed three times with phosphate buffer solution and fixed with FACS Lysing solution (BD Biosciences; Franklin Lakes, NJ, USA). The respective isotypes (FITC, PE, and APC) were used as controls. Cell fluorescence was measured by flow cytometry using FACSVerse (BD Biosciences; Franklin Lakes, NJ, USA), and a total of 3 × 10^6^ events were analyzed using the Suits software (BD Biosciences; Franklin Lakes, NJ, USA). CD34^+^/VEGFR2^+^ cells and CD34^+^/CD133^+^/VEGFR2^+^ cells were considered CACs. They were calculated as a percentage of VEGFR2^+^ cells and CD133^+^/VEGFR2^+^ cells in the CD34^+^ gate, respectively. Intraclass correlation coefficient was 0.80 for CD34^+^/VEGFR2^+^ cells and 0.90 for CD34^+^/CD133^+^/VEGFR2^+^ cells.

### 2.11. Gelatin Zymography

Gelatinolytic activity of serum MMP-2 and MMP-9 was measured using the gelatin zymography technique. Quantification of serum protein was determined by the Lowry method [21]. Proteins were electrophoresed through a 9% polyacrylamide gel copolymerized with gelatin (2 mg/mL, type A from porcine skin; Sigma-Aldrich, St. Louis, MO, USA) and a 4% polyacrylamide stacking gel. The gels were washed with 2.5% Triton X-100 and incubated for 24 h at 37°C in activation buffer (10 mM Tris buffer, pH 7.5, with 5 mM CaCl_2_ and 1 *μ*M ZnCl_2_) in order to verify the activity of the enzyme. After incubation, the gels were stained with a solution containing 30% methanol, 10% acetic acid, and 0.05% Coomassie brilliant blue (R-250; Sigma-Aldrich, St. Louis, MO, USA). Gelatinolytic activities were defined as transparent bands against the dark blue background. Zymograms were digitally scanned. The band intensities were measured using Scion Image (Scion Corporation; Frederick, MD, USA) and expressed as a ratio to the internal standard. Fetal bovine serum was used in each gel as a molecular weight standard for gelatinases and as an internal standard to correct for intergel variability.

### 2.12. Statistical Analysis

Data distribution was determined through the Shapiro-Wilk test and homogeneity of variances by Levene's test. A total sample size of 8 subjects was necessary to detect differences of 5% between groups (group main effect), considering a two-way ANOVA *P* value of 0.05 and power of 0.80. Unpaired Student's *t*-test was performed to identify significance between group differences in all normally distributed variables. When distributional assumption of normality was not met, the statistical inference was obtained using the Mann-Whitney *U* test, an equivalent nonparametric test. A chi-square test was used to analyze categorical variables. Two-way ANOVA was used to compare the variables before and after exercise or nonexercise session between the groups, followed by Fisher post hoc test in case of significant interaction, group, and/or moment effect. All the concentration values of adhesion molecules were multiplied by a constant 100. Outliers were considered as the mean ± three times the standard deviation and were excluded from the analyses. Significance was accepted at the 0.05 level.

## 3. Results

The anthropometric, clinical, and biochemical profiles of healthy controls and subjects with early MetS are presented in Table 1. As expected, body mass, body mass index (BMI), body fat, waist circumference, blood pressure, lipid profile, and glucose profile were significantly different between the healthy and MetS groups. There was no difference between groups regarding gender, age, absolute peak oxygen consumption (VO_2peak_), and total leukocyte count.

**Figure 1:**
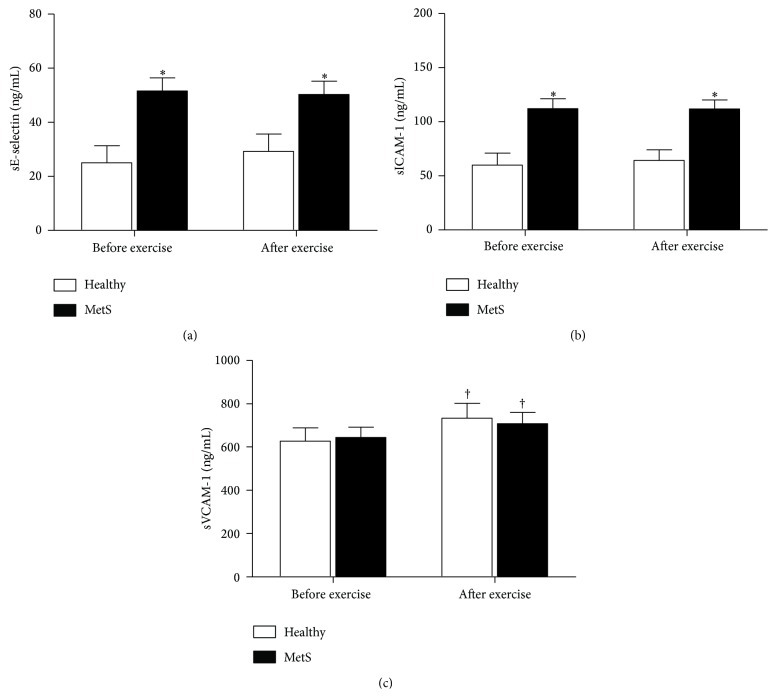
Adhesion molecules in healthy controls and subjects with MetS before and after exercise. sE-selectin: soluble endothelial selectin; sICAM-1: soluble intercellular adhesion molecule-1; sVCAM-1: soluble vascular cell adhesion molecule-1. ^*∗*^ *P* ≤ 0.01 versus healthy controls; ^†^ *P* ≤ 0.03 versus before exercise.

Figure 1 shows the levels of adhesion molecules (sE-selectin, sICAM-1, and sVCAM-1) before and after exercise in healthy controls and subjects with early MetS. Levels of sE-selectin and sICAM-1 were 106% and 87% higher in subjects with early MetS when compared with healthy controls (*P* < 0.01; Figure 1) at baseline. After exercise, the levels of these molecules were still higher than in healthy controls (*P* ≤ 0.01). sVCAM-1 levels were similar between groups at baseline (*P* = 0.83) and after exercise (*P* = 0.76). However, both groups presented increased sVCAM-1 levels after exercise (*P* ≤ 0.03).

**Figure 2:**
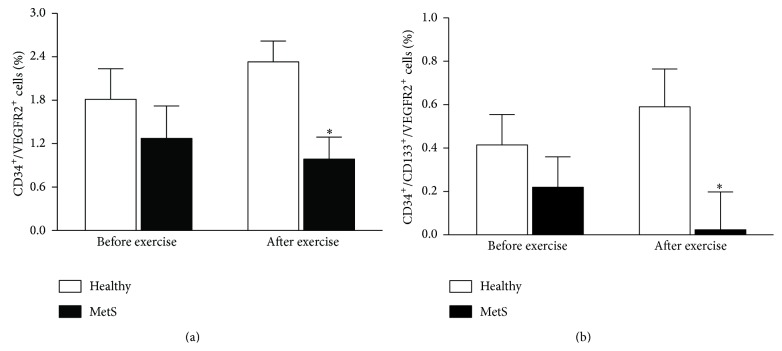
Circulating angiogenic cells ((a) CD34^+^/VEGFR2^+^ cells; (b) CD34^+^/CD133^+^/VEGFR2^+^ cells) before and after exercise in healthy controls and subjects with MetS. ^*∗*^ *P* = 0.02 versus healthy controls.

Regarding the flow cytometry data (Figure 2), no difference was found at baseline in either CD34^+^/VEGFR2^+^ cells (*P* = 0.31; Figure 2(a)) or CD34^+^/CD133^+^/VEGFR2^+^ cells (*P* = 0.39; Figure 2(b)) between groups. Subjects with early MetS showed a lower number of CD34^+^/VEGFR2^+^ cells (*P* = 0.02) and CD34^+^/CD133^+^/VEGFR2^+^ cells (*P* = 0.02) than healthy controls after exercise.

Subjects with early MetS presented higher levels of MMP-9 at baseline (*P* = 0.04) and after exercise (*P* = 0.02) when compared to healthy controls (Table 2). Although G-CSF have increased similarly in both groups after exercise (*P* < 0.05), no differences were observed in the serum concentration of all angiogenic factors between groups before and after exercise (*P* > 0.05; Table 2).

**Figure 3:**
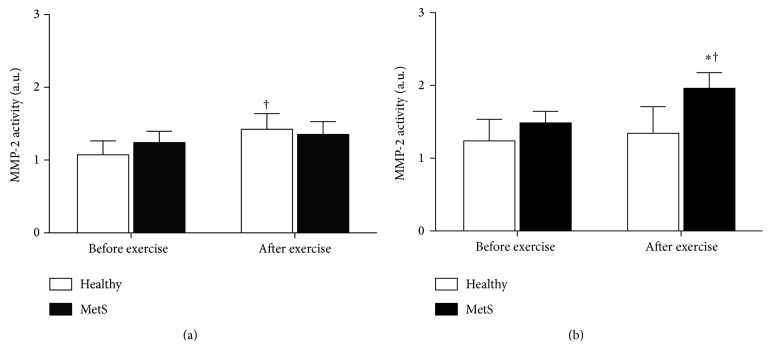
MMP-2 (a) and MMP-9 (b) activities before and after exercise in healthy controls and subjects with MetS. MMP-2: matrix metalloproteinase-2; MMP-9: matrix metalloproteinase-9. ^*∗*^ *P* < 0.05 versus healthy controls; ^†^ *P* ≤ 0.01 versus before exercise.

Figure 3 shows the MMP-2 and MMP-9 activities before and after exercise sessions in healthy controls and subjects with early MetS. At baseline, no differences were found in MMP-2 and MMP-9 activities between groups (*P* > 0.05; Figures 3(a) and 3(b), resp.). After exercise, only healthy controls had increased MMP-2 activity after exercise (*P* < 0.01; Figure 3(a)). In addition, subjects with early MetS presented an increase in MMP-9 activity after exercise (*P* = 0.01; Figure 3(b)), which was different between groups (*P* < 0.05; Figure 3(b)).

There was no difference in all variables between moments in nonexercise session (*P* > 0.05; data not shown).

## 4. Discussion

Our study hypothesized that subjects with early MetS already presented an impaired endothelial function along with an altered response of angiogenic factors, CACs, and MMPs to exercise. This study presented four novel findings: (1) subjects with early MetS presented higher levels of adhesion molecules and MMP-9 and similar levels of CD34^+^/VEGFR2^+^ and CD34^+^/CD133^+^/VEGFR2^+^ cells at baseline compared with healthy controls; (2) subjects with early MetS presented a lower number of CD34^+^/VEGFR2^+^ and CD34^+^/CD133^+^/VEGFR2^+^ cells after exercise; (3) healthy controls presented increased MMP-2 activity, while subjects with early MetS presented higher MMP-9 activity after exercise; and (4) no differences were observed in angiogenic factors between groups before and after exercise.

Adhesion molecules play a critical role during inflammatory responses by mediating the interaction of leukocytes to endothelial cells and, subsequently, their migration into perivascular tissues [22]. Studies have associated high levels of cell adhesion molecules with endothelial dysfunction and the development of atherosclerosis [23]. It was also demonstrated that subjects with early MetS already present an increased brachial artery time to peak diameter and a reduced shear rate-adjusted flow-mediated dilation [24], which may represent independent markers of endothelial function in subjects with early MetS. The present study corroborated these findings, showing that subjects with early MetS presented increased baseline levels of sE-selectin and sICAM-1 and, consequently, early endothelial dysfunction. As observed for adhesion molecules, the levels of MMP-9 seemed to be higher in subjects with early MetS, while MMPs activities were not. Some studies have demonstrated that increased baseline level of MMP-9 is related to atherothrombotic risk in subjects with cardiometabolic diseases [25] and in healthy subjects [26].

Differences between the groups regarding CD34^+^/VEGFR2^+^ and CD34^+^/CD133^+^/VEGFR2^+^ cells were observed after exercise. This fact could be partially explained by an elevated proinflammatory state [27]. In fact, levels of sE-selectin and sICAM-1 were still increased after exercise in subjects with early MetS, which contributes to apoptosis or loss of CACs functionality. Acute exercise induces a transient inflammatory response through the increase in several cytokines such as interleukin-6, tumor necrosis factor-*α*, C-reactive protein [14], and nuclear factor kappa B [28] and oxidative stress [29] in healthy subjects and in subjects with coronary artery disease [30]. In contrast, chronic repetitive exercise, that is, physical training, induces the development of an adaptation to the acute stress of exercise bouts [31] and reduces proinflammatory cytokine basal levels while inducing the expression of antioxidant and anti-inflammatory variables in the vessel [30]. These factors may directly inhibit the development of atherosclerosis and, consequently, diminish the risk of cardiovascular events [32].

Previous studies have shown that chronic exercise increases the number of CACs in subjects with MetS [33, 34]. However, these subjects presented established diseases, such as hypertension, diabetes, and coronary artery disease, and were under the effect of different medications. Therefore, the results of those studies could be biased by the presence of cardiometabolic diseases or the pleiotropic effects of the drugs. To our knowledge, the present study is the first to investigate the number of CACs after one bout of exercise in subjects with early MetS, that is, free of overt disease or pharmacological treatment.

An exercise bout increased MMP-2 activity in healthy controls and MMP-9 activity in subjects with early MetS. A previous study has already demonstrated opposite behaviors between serum MMP-2 and MMP-9 in critical limb ischemia patients [35]. It was also shown that expression of MMP-2 and MMP-9 on CACs surface plays a role in their invasive capacity and the guidance of circulating endothelial cells (mature or progenitor) to ischemic regions. Exercise is a physiological stimulus, which increases local production and release of growth factors and chemoattractant cytokines [36, 37]. These factors are able to activate MMPs, causing CACs mobilization from bone marrow to peripheral circulation [36–38]. It was shown that G-CSF is able to increase MMP-2 activity in human trophoblast cell line, through activation of PI3K/Akt and Erk signaling pathways [38]. Although subjects with early MetS also presented increased levels of G-CSF after exercise, they failed to increase MMP-2 activity and release CACs to peripheral blood. Other studies are necessary to address possible mechanisms that activate MMP-2 in MetS.

In addition, MMP-9 is released from skeletal muscles into the circulation as a response to proinflammatory conditions, contributing to the disruption of atherosclerotic plaques [39]. It was already shown that concentration and activity of MMP-9 increased during exercise in healthy subjects, returning to baseline levels at the end of exercise [15]. Our results corroborated these findings, showing that there is no difference between the pre- and postexercise moments in healthy controls. On the other hand, it seems that subjects with early MetS exhibited a sustained or late increase in MMP-9 activity after exercise. MMP-9 high levels and activity have been considered independent predictors for the development of coronary artery diseases [16] and are associated with increased cardiovascular risk in subjects with MetS [40].

CACs and MMP-9 may be considered independent biomarkers for endothelial function, predicting the onset of MetS-related diseases. Although the activation of proteinases, such as MMP-9, increases CACs mobilization from the bone marrow quiescent niche [41], the increase in MMP-9 activity was not enough to increase CACs in subjects with early MetS. It is conceivable that MMP-9 high activity, after exercise in subjects with early MetS, may have led to a transitory increase in CACs mobilization from the bone marrow to peripheral blood followed by an increase in CACs consumption by the impaired endothelium in subjects with early MetS. However, other specific studies are necessary to confirm these hypotheses. Moreover, there are other molecules and conditions, such as nitric oxide and oxidative stress, which could influence the number of CACs after exercise. NO is already known as a potent stimulus of CACs mobilization. A lower bioavailability of serum NO after exercise would be a mechanism that may explain the diminished levels of CACs in subjects with early MetS. It was also shown that antioxidative enzyme levels are reduced [42], while oxidative stress is increased in subjects with MetS [42] or after acute exercise [29].

The present study should be interpreted considering some limitations. First, anti-CD34, anti-VEGFR2, and anti-CD133 antibodies were used to quantify CACs by flow cytometry. Currently, there is no gold-standard marker for characterization of CACs. This point makes it difficult to standardize and compare the quantification of CACs among the different published studies. However, CD34^+^/VEGFR2^+^ and CD34^+^/CD133^+^/VEGFR2^+^ are most frequently used for their identification because the level of circulating CD34^+^/VEGFR2^+^ cells predicts the occurrence of cardiovascular events and death, which may help to identify patients at increased cardiovascular risk [43]. Second, we used men and women in the same analysis. To counter this limitation, the groups were matched for sex differences and all the women were evaluated in the follicular phase of the menstrual cycle. A third potential limitation was the absence of differences in CACs number at baseline between the groups. Some studies have demonstrated that subjects with cardiometabolic diseases present reduced baseline levels of CD34^+^/VEGFR2^+^ cells [11] when compared with healthy controls. However, the current population with MetS is free of overt disease or pharmacological treatment, and these factors are known to alter the results [12, 13, 44].

In conclusion, despite being free of established chronic diseases and pharmacological treatment, the subjects with MetS already presented an early impairment of endothelial function, as shown by increased baseline levels of sE-selectin, sICAM-1, and MMP-9. In addition, subjects with early MetS already exhibited an impaired response to exercise in terms of CACs and MMP-9 activity. The analysis of these biomarker changes could be potentially useful to develop preventive measures before the onset of MetS-related diseases.

## Figures and Tables

**Table 1:** Selected subject characteristics.

Variable	Healthy	MetS	*P* value
Number	9	15	—
Age, yr	33 ± 3	37 ± 2	0.23
Sex, M/W	6/3	12/3	0.47
Body mass, kg	71 ± 4	93 ± 4	<0.01
BMI, kg·m^−2^	23 ± 1	31 ± 1	<0.01
Body fat, %	26 ± 2	36 ± 2	<0.01
Waist circumference, cm	79 ± 3	102 ± 3	<0.01
Systolic BP, mmHg	117 ± 2	126 ± 3	<0.01
Diastolic BP, mmHg	75 ± 2	84 ± 2	<0.01
VO_2peak_, L·min^−1^	2.3 ± 0.2	2.2 ± 0.3	0.74
Total cholesterol, mmol·L^−1^	4.4 ± 0.2	5.6 ± 0.2	<0.01
LDL cholesterol, mmol·L^−1^	2.7 ± 0.2	3.6 ± 0.2	<0.01
HDL cholesterol, mmol·L^−1*∗*^	1.3 ± 0.5	1 ± 0.2	<0.01
Triglycerides, mmol·L^−1^	0.7 ± 0.1	2.1 ± 0.2	<0.01
Glucose, mmol·L^−1^	4.8 ± 0.1	5.5 ± 0.2	<0.01
Insulin, *μ*IU·mL^−1*∗*^	7.4 ± 2.5	13.8 ± 12.5	<0.01
HOMA-IR^*∗*^	1.5 ± 1.0	3.0 ± 3.5	<0.01
Total leukocytes count, 10^3^·mm^−3^	6.2 ± 0.8	6.8 ± 0.4	0.44

Values are means ± SE or ^*∗*^medians ± interquartile range. M: men; W: women; BMI: body mass index; BP: blood pressure; LDL: low-density lipoprotein; HDL: high-density lipoprotein; HOMA-IR: homeostasis model of insulin resistance.

**Table 2:** Serum concentration of MMP-9 and angiogenic factors before and after exercise.

Variables	Healthy		MetS	
	Before exercise	After exercise	Before exercise	After exercise
MMP-9 (ng·mL^−1^)	1.6 ± 0.3	1.8 ± 0.3	2.3 ± 0.2^*∗*^	2.6 ± 0.2^*∗*^
VEGF (pg·mL^−1^)	225.1 ± 65.3	241.6 ± 65.8	146.6 ± 46.1	155.5 ± 46.5
G-CSF (pg·mL^−1^)	11.8 ± 3.7	15.8 ± 4.6^†^	17.3 ± 2.9	21.3 ± 3.6^†^
GM-CSF (pg·mL^−1^)	1.3 ± 1.2	1.6 ± 1.1	2.6 ± 0.9	3.3 ± 0.9

Values are means ± SE. Healthy: healthy controls; MetS: subjects with metabolic syndrome; MMP-9: metalloproteinase-9; VEGF: vascular endothelial growth factor; G-CSF: granulocyte-colony stimulating factor; GM-CSF: granulocyte macrophage-colony stimulating factor. ^*∗*^ *P* ≤ 0.04 versus healthy controls; ^†^ *P* < 0.05 versus before exercise.

## References

[1] World Health Organization (2014). *Cardiovascular Diseases*.

[2] Lakka H.-M., Laaksonen D. E., Lakka T. A. (2002). The metabolic syndrome and total and cardiovascular disease mortality in middle-aged men. *Journal of the American Medical Association*.

[3] Alberti K. G. M. M., Eckel R. H., Grundy S. M. (2009). Harmonizing the metabolic syndrome: a joint interim statement of the international diabetes federation task force on epidemiology and prevention; National heart, lung, and blood institute; American heart association; World heart federation; International atherosclerosis society; And international association for the study of obesity. *Circulation*.

[4] Nikolopoulou A., Kadoglou N. P. E. (2012). Obesity and metabolic syndrome as related to cardiovascular disease. *Expert Review of Cardiovascular Therapy*.

[5] Stoolman L. M. (1993). Adhesion molecules involved in leukocyte recruitment and lymphocyte recirculation. *Chest*.

[6] Chen X., Scholl T. O. (2014). Maternal biomarkers of endothelial dysfunction and preterm delivery. *PLoS ONE*.

[7] Gómez Rosso L., Benítez M. B., Fornari M. C. (2008). Alterations in cell adhesion molecules and other biomarkers of cardiovascular disease in patients with metabolic syndrome. *Atherosclerosis*.

[8] Fernandes T., Nakamuta J. S., Magalhães F. C. (2012). Exercise training restores the endothelial progenitor cells number and function in hypertension: implications for angiogenesis. *Journal of Hypertension*.

[9] Spigoni V., Picconi A., Cito M. (2012). Pioglitazone improves in vitro viability and function of endothelial progenitor cells from individuals with impaired glucose tolerance. *PLoS ONE*.

[10] Rossi F., Bertone C., Montanile F. (2010). HDL cholesterol is a strong determinant of endothelial progenitor cells in hypercholesterolemic subjects. *Microvascular Research*.

[11] Jialal I., Devaraj S., Singh U., Huet B. A. (2010). Decreased number and impaired functionality of endothelial progenitor cells in subjects with metabolic syndrome: implications for increased cardiovascular risk. *Atherosclerosis*.

[12] Sun J.-Y., Zhai L., Li Q.-L. (2013). Effects of ACE inhibition on endothelial progenitor cell mobilization and prognosis after acute myocardial infarction in type 2 diabetic patients. *Clinics*.

[13] Dimmeler S., Aicher A., Vasa M. (2001). HMG-CoA reductase inhibitors (statins) increase endothelial progenitor cells via the PI 3-kinase/Akt pathway. *Journal of Clinical Investigation*.

[14] Hamer M., Steptoe A. (2012). Vascular inflammation and blood pressure response to acute exercise. *European Journal of Applied Physiology*.

[15] Rullman E., Olsson K., Wågsäter D., Gustafsson T. (2013). Circulating MMP-9 during exercise in humans. *European Journal of Applied Physiology*.

[16] Ferroni P., Basili S., Martini F. (2003). Serum metalloproteinase 9 levels in patients with coronary artery disease: a novel marker of inflammation. *Journal of Investigative Medicine*.

[17] Rummens J. L., Daniëls A., Dendale P. (2012). Suppressed increase in blood endothelial progenitor cell content as result of single exhaustive exercise bout in male revascularised coronary artery disease patients. *Acta Clinica Belgica*.

[18] Matthews D. R., Hosker J. P., Rudenski A. S., Naylor B. A., Treacher D. F., Turner R. C. (1985). Homeostasis model assessment: insulin resistance and *β*-cell function from fasting plasma glucose and insulin concentrations in man. *Diabetologia*.

[19] Skogstrand K., Thorsen P., Nørgaard-Pedersen B., Schendel D. E., Sørensen L. C., Hougaard D. M. (2005). Simultaneous measurement of 25 inflammatory markers and neurotrophins in neonatal dried blood spots by immunoassay with xMAP technology. *Clinical Chemistry*.

[20] Carson R. T., Vignali D. A. A. (1999). Simultaneous quantitation of 15 cytokines using a multiplexed flow cytometric assay. *Journal of Immunological Methods*.

[21] Lowry O. H., Rosebrough N. J., Farr A. L., Randall R. J. (1951). Protein measurement with the Folin phenol reagent. *The Journal of Biological Chemistry*.

[22] Davies M. J., Gordon J. L., Gearing A. J. H. (1993). The expression of the adhesion molecules ICAM-1, VCAM-1, PECAM, and E-selectin in human atherosclerosis. *Journal of Pathology*.

[23] Hwang S.-J., Ballantyne C. M., Sharrett A. R. (1997). Circulating adhesion molecules VCAM-1, ICAM-1, and E-selectin in carotid atherosclerosis and incident coronary heart disease cases: the Atherosclerosis Risk In Communities (ARIC) study. *Circulation*.

[24] Fernandes I. A., Sales A. R. K., Rocha N. G., Silva B. M., Vianna L. C., Da Nóbrega A. C. L. (2014). Preserved flow-mediated dilation but delayed time-to-peak diameter in individuals with metabolic syndrome. *Clinical Physiology and Functional Imaging*.

[25] Tayebjee M. H., Nadar S. K., MacFadyen R. J., Lip G. Y. H. (2004). Tissue inhibitor of metalloproteinase-1 and matrix metalloproteinase-9 levels in patients with hypertension: relationship to tissue Doppler indices of diastolic relaxation. *American Journal of Hypertension*.

[26] Sundström J., Vasan R. S. (2006). Circulating biomarkers of extracellular matrix remodeling and risk of atherosclerotic events. *Current Opinion in Lipidology*.

[27] Zaldivar F., Wang-Rodriguez J., Nemet D. (2006). Constitutive pro- and anti-inflammatory cytokine and growth factor response to exercise in leukocytes. *Journal of Applied Physiology*.

[28] Balan M., Locke M. (2011). Acute exercise activates myocardial nuclear factor kappa B. *Cell Stress and Chaperones*.

[29] Bogdanis G. C., Stavrinou P., Fatouros I. G. (2013). Short-term high-intensity interval exercise training attenuates oxidative stress responses and improves antioxidant status in healthy humans. *Food and Chemical Toxicology*.

[30] Lara Fernandes J., Serrano C. V., Toledo F. (2011). Acute and chronic effects of exercise on inflammatory markers and B-type natriuretic peptide in patients with coronary artery disease. *Clinical Research in Cardiology*.

[31] da Nobrega A. C. L. (2005). The subacute effects of exercise: concept, characteristics, and clinical implications. *Exercise and Sport Sciences Reviews*.

[32] Green D. J., O'Driscoll G., Joyner M. J., Cable N. T. (2008). Exercise and cardiovascular risk reduction: time to update the rationale for exercise?. *Journal of Applied Physiology*.

[33] Sonnenschein K., Horváth T., Mueller M. (2011). Exercise training improves in vivo endothelial repair capacity of early endothelial progenitor cells in subjects with metabolic syndrome. *European Journal of Cardiovascular Prevention and Rehabilitation*.

[34] Fernández J. M., Rosado-Álvarez D., Grigoletto M. E. D. S. (2012). Moderate-to-high-intensity training and a hypocaloric Mediterranean diet enhance endothelial progenitor cells and fitness in subjects with the metabolic syndrome. *Clinical Science*.

[35] Teraa M., Sprengers R. W., Westerweel P. E. (2013). Bone marrow alterations and lower endothelial progenitor cell numbers in critical limb ischemia patients. *PLoS ONE*.

[36] Chang E., Paterno J., Duscher D. (2015). Exercise induces stromal cell-derived factor-1alpha-mediated release of endothelial progenitor cells with increased vasculogenic function. *Plastic and Reconstructive Surgery*.

[37] Kruger K., Klocke R., Kloster J., Nikol S., Waltenberger J., Mooren F. C. (2014). Activity of daily living is associated with circulating CD34^+^/KDR^+^ cells and granulocyte colony-stimulating factor levels in patients after myocardial infarction. *Journal of Applied Physiology*.

[38] Furmento V., Marino J., Blank V., Roguin L. (2014). The granulocyte colony-stimulating factor (G-CSF) upregulates metalloproteinase-2 and VEGF through PI3K/Akt and Erk1/2 activation in human trophoblast Swan 71 cells. *Placenta*.

[39] Newby A. C. (2008). Metalloproteinase expression in monocytes and macrophages and its relationship to atherosclerotic plaque instability. *Arteriosclerosis, Thrombosis, and Vascular Biology*.

[40] Cicero A., Derosa G., Manca M., Bove M., Borghi C., Gaddi A. (2007). Vascular remodeling and prothrombotic markers in subjects affected by familial combined hyperlipidemia and/or metabolic syndrome in primary prevention for cardiovascular disease. *Endothelium*.

[41] Heissig B., Hattori K., Dias S. (2002). Recruitment of stem and progenitor cells from the bone marrow niche requires MMP-9 mediated release of Kit-ligand. *Cell*.

[42] Furukawa S., Fujita T., Shimabukuro M. (2004). Increased oxidative stress in obesity and its impact on metabolic syndrome. *The Journal of Clinical Investigation*.

[43] Werner N., Kosiol S., Schiegl T. (2005). Circulating endothelial progenitor cells and cardiovascular outcomes. *The New England Journal of Medicine*.

[44] Endtmann C., Ebrahimian T., Czech T. (2011). Angiotensin II impairs endothelial progenitor cell number and function in vitro and in vivo: implications for vascular regeneration. *Hypertension*.

